# Attention allocation in complementary joint action: How joint goals affect spatial orienting

**DOI:** 10.3758/s13414-023-02779-1

**Published:** 2023-09-08

**Authors:** Laura Schmitz, Basil Wahn, Melanie Krüger

**Affiliations:** 1 Institute of Sports Science, Leibniz University Hannover, Hannover, Germany; 2 Department of Neurology, University Medical Center Hamburg-Eppendorf, Hamburg, Germany; 3 Institute of Educational Research, Ruhr University Bochum, Bochum, Germany

**Keywords:** Joint action, Attentional orienting, Joint goals, Social inhibition of return, Perception-action coupling, Action observation, Complementary actions

## Abstract

When acting jointly, individuals often attend and respond to the same object or spatial location in complementary ways (e.g., when passing a mug, one person grasps its handle with a precision grip; the other receives it with a whole-hand grip). At the same time, the spatial relation between individuals’ actions affects attentional orienting: one is slower to attend and respond to locations another person previously acted upon than to alternate locations (“social inhibition of return”, social IOR). Achieving joint goals (e.g., passing a mug), however, often requires complementary return responses to a co-actor’s previous location. This raises the question of whether attentional orienting, and hence the social IOR, is affected by the (joint) goal our actions are directed at. The present study addresses this question. Participants responded to cued locations on a computer screen, taking turns with a virtual co-actor. They pursued either an individual goal or performed complementary actions with the co-actor, in pursuit of a joint goal. Four experiments showed that the social IOR was significantly modulated when participant and co-actor pursued a joint goal. This suggests that attentional orienting is affected not only by the spatial but also by the social relation between two agents’ actions. Our findings thus extend research on interpersonal perception-action effects, showing that the way another agent’s perceived action shapes our own depends on whether we share a joint goal with that agent.

**Supplementary Information:**

The online version contains supplementary material available at 10.3758/s13414-023-02779-1.

## Introduction

Successful social interaction often requires individuals to act upon the same object or spatial location in complementary ways (e.g., Knoblich et al., 2011; Richardson et al., 2015; Sartori & Betti, 2015; Sebanz et al., 2006). Consider, for example, two friends having tea together. One friend grasps a tea mug by its handle with a precision grip and places it on the table, where it is picked up by the other friend with a complementary whole-hand grip. Or consider two friends painting a landscape together. Since they are lacking green paint, one friend pours yellow paint into a bucket and the other then adds the complementary blue paint, jointly creating green. In these and countless other social interactions, individuals need to overcome their tendency to imitate an observed action (Brass et al., 2009; Hamilton, 2015; Heyes, 2011) – a tendency supported by automatic resonance processes in the observer’s motor system (for a review, see Rizzolatti & Sinigaglia, 2010) – and instead perform a complementary action to achieve a particular joint goal, such as passing a mug or painting in green (on joint/shared goals, see Butterfill, 2012; Sacheli et al., 2015; Vesper et al., 2010). To this end, the human motor system flexibly shifts from its automatic imitative to a complementary action tendency early on (Sartori et al., 2011, 2012, 2013). At the brain level, complementary joint actions elicit additional cortical motor activation compared to imitative joint actions (Era et al., 2018; Kokal et al., 2009; Ménoret et al., 2014; Naeem et al., 2012a, b; Newman-Norlund et al., 2007, 2008), presumably because two non-identical (observed and executed) actions need to be integrated in the motor system (for reviews on brain activity in complementary joint action, see Bolt & Loehr, 2021, and Sartori & Betti, 2015). At the behavioral level, this mismatch between observed and (to be) executed actions can lead to visuo-motor interference effects in the form of slowed action execution and deviating movement kinematics (e.g., Brass et al., 2000; Kilner et al., 2003). Together, these findings demonstrate how the perception of others’ actions has the potential to shape our own actions at the brain and behavioral level.

Whether and to what extent our own actions are shaped by the perception of others’ actions depends on a variety of top-down factors such as social cues (Krishnan-Barman & Hamilton, 2019; Marsh et al., 2016; Wang & Hamilton, 2012, 2014), attentional focus (Longo et al., 2008; Wild et al., 2010), and experience (Catmur et al., 2007; Heyes et al., 2005). In the present study, we concentrate on another influencing factor, namely the goal our actions are directed at. Specifically, we will address the question of whether pursuing a *joint* goal with another agent shapes the way in which that agent’s action shapes our own. Previous research has already provided initial behavioral evidence to that effect (while neurophysiological evidence is still rare; but see recent work by Barchiesi et al., 2022, and Sacheli et al., 2022). For example, several behavioral studies showed that, if the goal of a joint action requires the performance of two complementary actions, individuals will actually be faster to respond to a co-actor’s action with a different (yet socially appropriate!) action rather than with the identical action, thus reversing the otherwise typical visuo-motor interference effect (Betti et al., 2019; Newman-Norlund et al., 2007; Poljac et al., 2009; van Schie et al., 2008; also see Ocampo & Kritikos, 2010). Furthermore, studies by Sacheli and colleagues (2012, 2013) demonstrated that successful interactions (i.e., those in which participants achieved their joint goal) were characterized by the absence of visuo-motor interference. In these studies, two participants aimed for the joint goal of grasping a bottle-shaped object at the same time, with one of them (the “Leader”) applying either a precision or a whole-hand grip while the other (the “Follower”) needed to apply the respective complementary grip. To achieve the joint goal of grasping synchronicity, both Leaders and Followers managed to inhibit the tendency to imitate each other’s movements. Another study by Sacheli and colleagues (2018) demonstrated the influence of joint goals in a musical interaction context where participants either played a shared melody with or merely played alongside a partner. If participants’ actions were physically incongruent with the partner’s (e.g., grasp vs. point) and there was no joint goal, participants showed signs of visuo-motor interference. If a joint goal was present, however, there was no such interference. According to Sacheli et al., this indicates that participants represented their partner’s actions with respect to the joint goal (the shared melody). These results suggest that interference from another person’s observed incongruent actions can be circumvented if own and other’s actions are represented as complementary contributions to a joint goal (also see Clarke et al., 2019).

However, a study by Della Gatta and colleagues (2017) indicated that joint goals can also have the opposite effect, namely to increase visuo-motor interference between one’s own and another’s actions. In this study, participants were asked to draw either circles or lines while observing another individual drawing either circles or lines. Participants either performed the drawing task in parallel with the co-actor or they shared a joint goal. When a joint goal was present and participants drew lines while observing the co-actor drawing circles, their drawing trajectories showed stronger interference than participants who did not share a joint goal with the co-actor. According to della Gatta et al., this suggests that participants represented the joint goal (drawing a circle *and* a line) motorically, almost as if they were performing the two actions with their own two hands. Thus, the *intra*personal motor coupling typically observed in bimanual coordination (Franz et al., 1991) emerged here in the form of *inter*personal coupling (consistent with the account that coordination within and between individuals relies on similar processes (e.g., Fine & Amazeen, 2011; Richardson et al., 2007; Schmidt et al., 1998; Schmidt & Richardson, 2008)).

Taken together, previous research on the interplay between perception and action suggests that the way our own actions are shaped by the perception of others’ (preceding or concurrent) actions also depends on how we represent the relation between own and others’ actions. Do we represent the actions as *independent*, with each directed towards an *individual goal*, or as *complementary* and directed towards a *joint goal*? The aim of the present study was to further examine the impact of joint goals on interpersonal perception-action effects. Whereas previous studies focused on how action observation influences action execution in cases where two individuals perform actions with the same or different *kinematic parameters* (e.g., precision vs. whole-hand grip (Newman-Norlund et al., 2007; Ocampo & Kritikos, 2010; Poljac et al., 2009; Sacheli et al., 2012, 2013; van Schie et al., 2008), grasp vs. point (Sacheli et al., 2018), line vs. circle (Della Gatta, 2017)), the present study focuses on whether two individuals perform actions to the same or different *spatial locations*. We chose this focus because in many of the joint actions that require individuals to perform complementary actions with *different* kinematic parameters (e.g., handing over a mug by its handle and receiving it with a whole-hand grip), these actions are typically directed towards the *same* object or spatial location (e.g., a mug). This focus is of particular interest since the spatial relation between individuals’ actions has been shown to affect their attentional and response behavior – an action observation phenomenon known as “social inhibition of return” (social IOR).

The social IOR, first reported by Welsh et al. (2005), shows that individuals take longer to prepare and initiate a reaching movement to the same location previously acted upon by a co-actor compared to an alternate location that has not been acted upon (for a recent review, see Cole et al., 2019). That is, after having observed a co-actor reach to one of two targets, it takes you longer to return to the co-actor’s target compared to reaching to the opposite target. This interpersonal perception-action effect is known as the *social* inhibition of return because there is also a *non-social,* intrapersonal version of the effect. The original IOR, first described by Posner and Cohen in 1984, refers to the phenomenon that individuals take longer to return to a location just attended compared to a different location (also see Posner et al., 1985; for a review, see Klein, 2000). This “inhibitory aftereffect” (Klein, 2000) occurs *after* an initial facilitation effect: Initially, targets at the previously attended location are detected faster and then, after ~225 ms (Klein, 2000), the facilitation turns into inhibition such that targets at that location are detected more slowly compared to different locations (e.g., Egeth & Yantis, 1997). These effects are explained in terms of the time course of attentional orienting: after an event (e.g., a movement) has occurred at a peripheral location, attention is first reflexively drawn toward that location (facilitation) but it then disengages and orients away such that responses to subsequent events at the same location are delayed (inhibition). This way, the IOR fulfills the function of inhibiting non-relevant information by creating a response bias away from recently attended locations, which in turn promotes orienting towards novel locations, thereby facilitating visual search behavior (Klein, 1988; but see Smith & Henderson 2011). When applying this search facilitator proposal to social interaction, it seems plausible that one should neither return to a location previously searched by oneself nor to a location searched by another individual (see Welsh et al., 2005). Accordingly, the IOR might fulfill a similar function between as within people.

However, looking at social interaction more broadly, many situations occur where returning to a co-actor’s location is in fact an appropriate behavior. Imagine yourself picking up a mug with a whole-hand grip, just after another person has used a precision grip to place the mug on the table. Or imagine pouring blue paint into a bucket, just after another person has poured in yellow paint. The latter example might not be a daily activity yet is mentioned here again because it serves as a background story for the present study – and it is representative for more common actions such as pouring milk into a cup after another person has poured in the tea. In these scenarios, returning to the location just acted upon by a co-actor is necessary to fulfill a joint goal, like passing a mug, painting in green or having tea with milk. In light of these and similar social interactions, the slowing of return responses does not seem beneficial. Thus, the following question arises: If two individuals share a joint goal that requires complementary actions at the same location, does this goal have an effect on the inhibitory process that would normally lead to slowed responding when one individual returns to the other’s location? Or to put it briefly, do joint goals shape the social IOR? This is the question we address in the present study. Note that previous research has looked at action goals and the social IOR, yet – to the best of our knowledge – the question of whether the social IOR is sensitive to *joint* goals remains to be addressed^1^. Specifically, a joint goal here refers to a goal that is shared among two (or more) agents and that requires (complementary) contributions from all agents involved (see Vesper et al., 2010).

### Present study

**Fig. 1:**
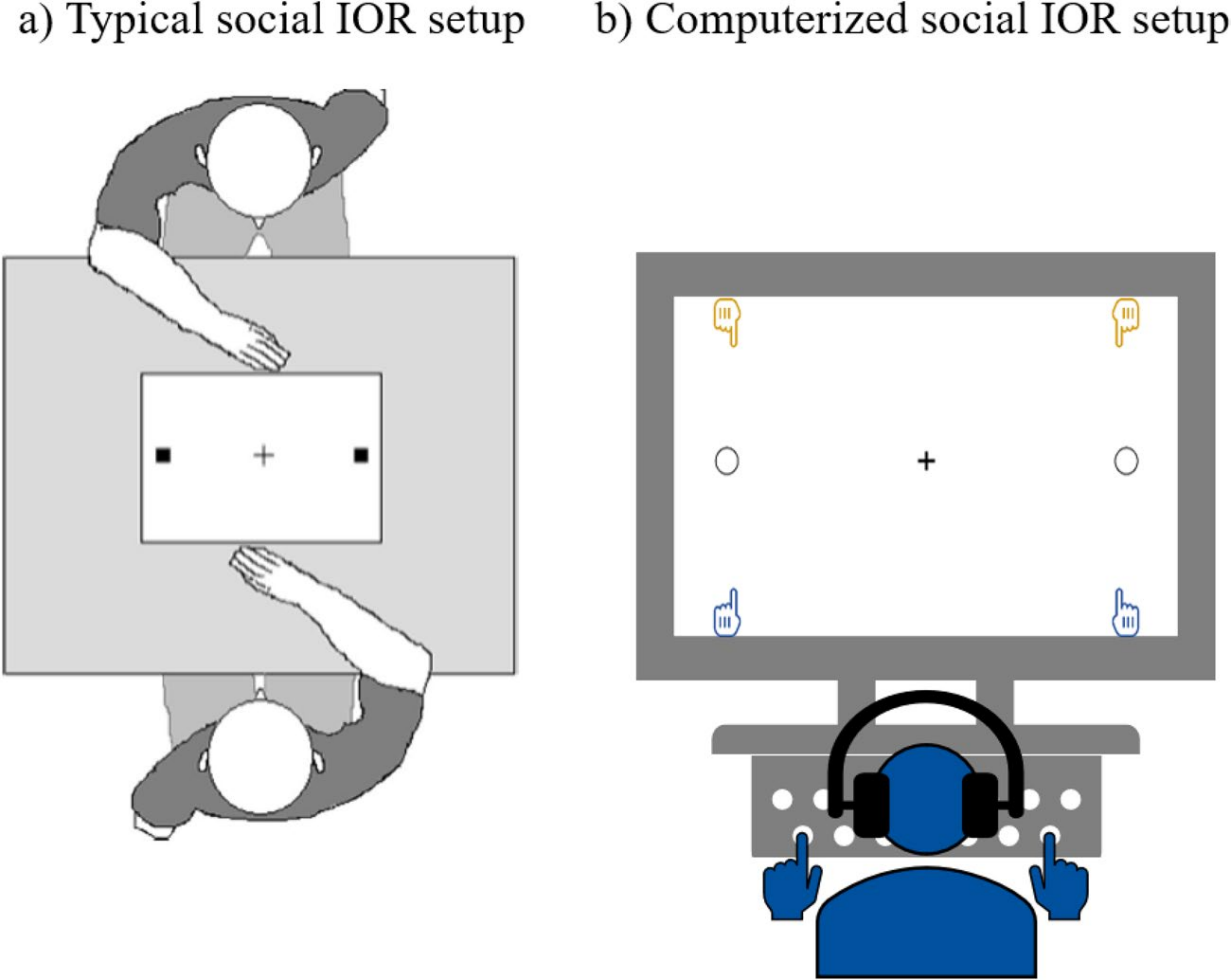
Sketch of the experimental setup used in (**a**) typical studies on the social IOR (reproduced with permission from Cole et al., 2019) and (**b**) the present study (not drawn to scale). Participants in the present study first observed a (yellow or blue) cue at one of the two target locations (empty circles) on the computer screen. Yellow meant that it was the co-actor’s turn and blue meant that it was the participant’s turn; turns always alternated. If it was their turn, participants pressed a key on the keyboard with their left or right hand (corresponding to the cue), causing the respective virtual hand on the screen to move to the left or right target. Once the target was reached, it turned blue. The virtual hand then returned to its starting position and the next trial began. For a detailed trial sequence, see Fig. 3

To address the above question, we designed a computerized version of the social IOR task.^2^ Participants responded to a cued location on a computer screen by pressing a left or right key on the keyboard, rather than reaching to a left or right location on a table as in the typical social IOR setup (see Fig. 1). Participants always responded in alternation with a so-called “virtual partner”; this partner was realized by a computer program. The partner will henceforth be simply referred to as “co-actor”. The participant’s key press triggered the motion of a virtual hand on the screen, moving to the left or right target and coloring it blue. If it was the co-actor’s turn, the co-actor’s virtual hand moved to the left or right target and colored it yellow.

To determine whether joint goals modulate the social IOR, we manipulated whether or not participants shared a joint goal with the co-actor. In the *Individual Goal condition*, participants’ goal was to color the target blue. In the *Joint Goal condition*, participants’ goal was to complement the co-actor’s response (by adding their own blue to the co-actor’s yellow) to color the target green. This joint goal was achieved if participants responded to the same target as the co-actor did in the previous trial.

**Fig. 2:**
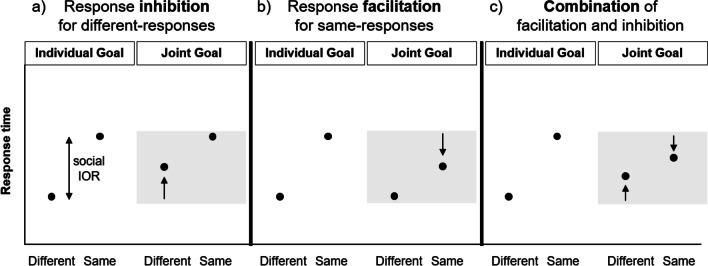
Hypothetical response patterns for the Joint Goal condition (highlighted in grey), displayed as a function of target location (Same = response to the co-actor’s previous location; Different = response to the opposite location). The predicted prolonged attention on the co-actor’s previous location is likely to inhibit responses to the opposite location (**a**), yet it could also facilitate responses to the same location (**b**), or do both (**c**). All three options would result in a smaller social inhibition of return (IOR; i.e., a smaller difference in response time between different- and same-responses) in the Joint Goal compared to the Individual Goal condition

Participants should be generally slower to initiate a response to the same target as the co-actor compared to the opposite target, reflecting the typical social IOR response pattern (Welsh et al., 2005). Would this pattern be different if they pursued a joint goal? Since the social IOR normally arises due to a particular attentional orienting behavior, a change in attentional orienting should also change the social IOR. To illustrate, assume that Jane and Joe have a joint goal which can be achieved by performing complementary actions at location X. Jane observes Joe responding to location X, knowing that the joint goal would be achieved if, in the next step, she also responded to location X (rather than location Y). Jane’s knowledge should make location X goal-relevant, which in turn should affect Jane’s orienting behavior such that her attention, which has reflexively shifted towards location X, remains there a little longer than usual. This would be in line with previous research on top-down attention showing that goal pursuit leads to intentional (e.g., Yantis, 2000) and unintentional (Vogt et al., 2010) allocation of spatial attention to goal-relevant events. Specifically, previous findings suggest that goal-relevant events can cause unintended *delayed disengagement* of attention (Vogt et al., 2010). Thus, in the present context, the pursuit of a joint goal could cause Jane’s attention to be allocated longer on the goal-relevant location X before disengaging. This prolonged attention allocation on X is likely to lead to a response inhibition (i.e., slower response times) for subsequent responses to Y (compare Vogt et al., 2010). Alternatively, it is also theoretically possible that it would lead to a response facilitation for X, or possibly even to both. All three options would effectively lead to a reduced social IOR (see Fig. 2). The following four experiments were performed to test whether joint goals affect attentional orienting and if so, whether this leads to response inhibition or facilitation.

## Experiment 1

### Method

#### Open practices and data availability

Below, we report how we determined our sample size, and we describe all data exclusions, all manipulations, and all dependent measures. All raw data and experimental stimuli are publicly available at the Open Science Framework (OSF) and can be accessed at: https://osf.io/4yfd2/?view_only=91af170d9953437bb9718a62ed5898af. Data were analyzed using R, version 4.0.5 (R Core Team, 2020) and the package ggplot, version 3.3.3 (Wickham, 2016). The study and analysis plan were preregistered via OSF (see https://osf.io/jehg6/?view_only=b5c0990d5bf6423da974adb5cb537acc).

#### Participants

We determined our target sample size based on previous research combined with an a priori power analysis. In particular, we relied on a study by Atkinson et al. (2018) that used a 2 × 2 within-subjects design and aimed to detect a potential interaction effect (just as in the present study; see below). Atkinson et al. argued for a sample size of 36, based on a meta-analysis of the social IOR by Cole et al. (2018). In addition, we ran a power analysis (alpha = 0.05, power = 0.80, two-tailed paired *t*-test) using G*Power (Faul et al., 2007) and determined that a sample size of 34 would allow us to detect a moderately sized interaction effect (Cohen’s *d* = 0.50; Cohen, 2013). Considering that we might have to exclude individual participants during analysis due to technical problems or other reasons (see below), we decided to collect a sample of 40 participants. Thus, we recruited 40 participants using the online recruitment service *Prolific* (https://www.prolific.co/). All participants had normal or corrected-to-normal vision and hearing and were fluent in English. Only participants whose performance in previous *Prolific* studies had been reliable (approval rates of at least 95%) were admitted to this study. Additionally, the use of a QWERTY or QWERTZ keyboard was a prerequisite for participation. All participants gave written informed consent and received monetary compensation for their participation (3 GBP for a total study duration of approx. 25 min). The study was conducted in line with the ethical principles of the Declaration of Helsinki and with the guidelines of the German Research Foundation (DFG) for the field of Psychology.

In Experiment 1, six participants were excluded from the analysis because they performed below 90% accuracy (see data exclusion criteria below), resulting in a final sample of 34 participants (*M*_*age*_ = 23.79 years, *SD*_*age*_ = 4.89 years; nine female, 25 male). Sixteen of these started with the Individual Goal condition and 18 with the Joint Goal condition. Thirty-two were right-handed, two were left-handed.

#### Stimuli and procedure

**Fig. 3:**
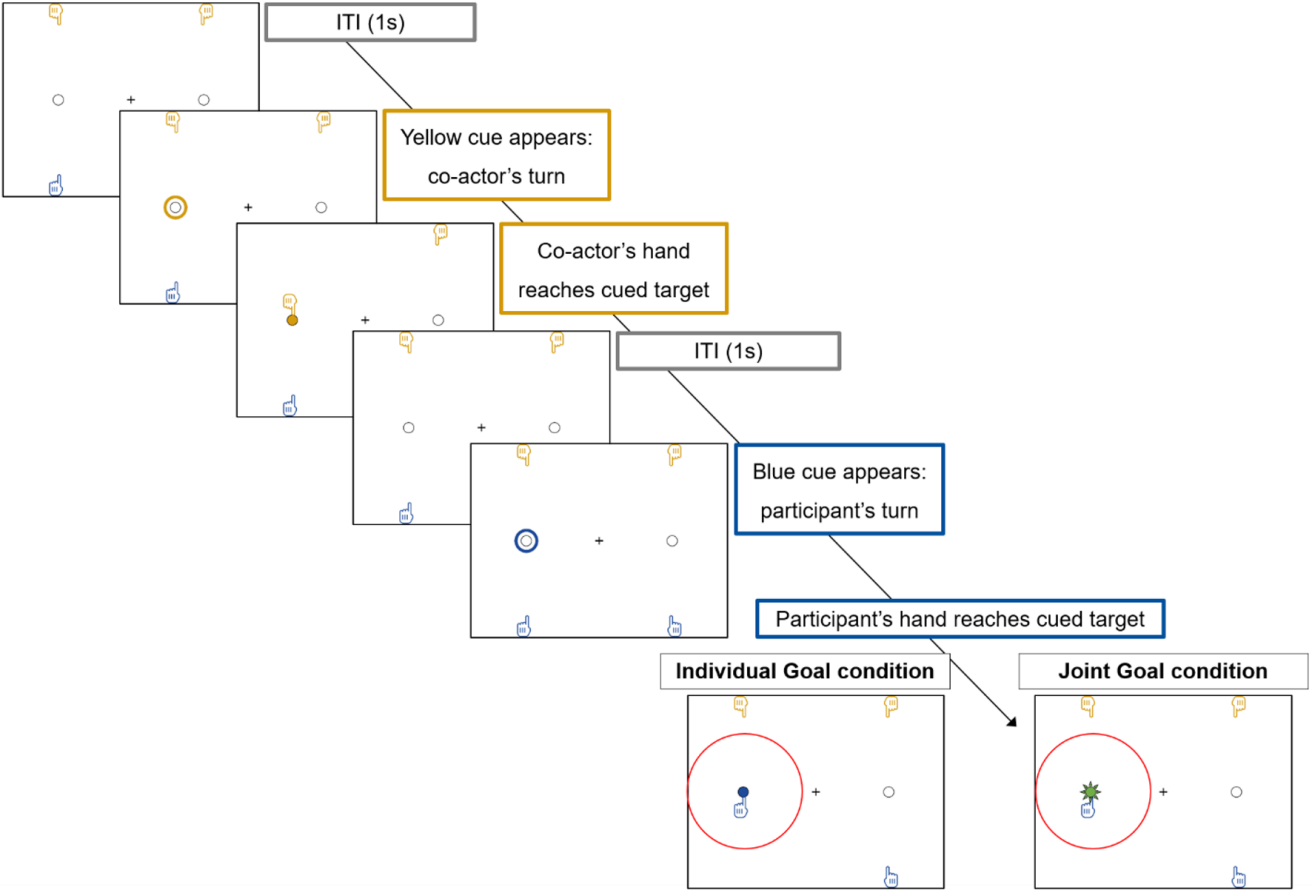
Exemplary trial sequence (Experiment 1): the co-actor (yellow) acts in the first trial (screens 2 and 3) and the participant (blue) acts in the next trial (screens 5 and 6). In this sequence, the participant responds to the same target location as the co-actor did in the previous trial. In the Individual Goal condition (left lower picture), participant’s response turns the target blue. In the Joint Goal condition (right lower picture), participant’s response turns the target green. (For illustration purposes only, the difference between Individual Goal and Joint Goal condition is circled in red.) The green color symbolizes that the joint goal has been reached, i.e., the participant’s blue color has been blended with the co-actor’s yellow color – an example of a complementary joint action. In trials in which participants responded to the opposite target (not depicted), the target turned blue in both conditions

The experiment was programed in *PsychoPy 3* (Peirce et al., 2019) and run online via *Pavlovia (*https://pavlovia.org/*)*. The task layout on the computer screen consisted of a central fixation cross and two target locations (empty circles with black outlines) to the left and right of the cross, respectively (Fig. 1). Participants were instructed to maintain fixation on the cross while performing the task. On the upper and lower edge of the screen, a pair of hands was depicted. The left lower and upper hands were vertically aligned with the left target; the right lower and upper hands were vertically aligned with the right target. The upper pair of hands was colored yellow, the lower one was colored blue. Each hand was depicted with its index finger sticking out, as if pointing towards the respective target. At the beginning of each trial, a cue (a colored outline) appeared around the left or right target location for 100 ms (see Fig. 3). The color of the outline indicated whose turn it was: yellow for the co-actor, blue for the participant. Participant and co-actor took turns, with the co-actor always acting first at the beginning of a new block. Participants were instructed to respond to the cue by pressing the corresponding letter key on the keyboard (W for left target, P for right target) with their left or right hand as quickly and accurately as possible. Participants kept their left index finger over the W key and their right index finger over the P key throughout the experiment. Upon key press, the corresponding hand on the screen moved to the target. If it was the co-actor’s turn, the co-actor’s hand started moving automatically. Once the hand reached the target (after 200 ms), the target turned blue (participant), yellow (co-actor) or green (joint goal; see below) for a duration of 500 ms during which the hand remained at the target (see Fig. 3). Note that the green target illumination was accompanied by a star-shaped green outline to increase its visual salience. Once the target illumination ended, the hand automatically returned to its starting position at the edge of the screen and the next cue appeared after an intertrial interval (ITI) of 1,000 ms. The co-actor’s response time was pre-programed and varied as a function of the participant’s preceding response location (same location: 350 ms, opposite location: 300 ms), in line with previous mean response times (e.g., Manzone et al., 2017).

At the beginning of the experiment, participants were informed that they would be playing a game with a virtual partner. In the Individual Goal condition, participants were instructed that their goal was to color the target in their own blue color by pressing the corresponding key. In the Joint Goal condition, participants were informed that, together with the virtual partner, they would be “working towards a joint goal,” which was to “turn the target green by blending your two colors (yellow and blue).” Participants were told that they would achieve the joint goal if they responded to the same target to which the partner had responded previously. Thus, whenever participants responded to the same target as the partner, the target turned green; the joint goal was achieved.

Note that the trial procedure was always the same (regardless of condition): First, a blue or yellow circle (“cue”) appeared around the left or right target, indicating *who* (participant or co-actor) should respond and *to which target* (left or right) they should respond. This meant that the participant’s cue could either appear around the same target to which the co-actor had responded in the previous trial or around the opposite target. Note that in both conditions, participants were instructed to always respond to the cue, i.e., they were not free to choose the target themselves. Consequently, in the Joint Goal condition, they could not decide whether to fulfill the joint goal or not; their response was contingent on the cue. The only differences between the Individual Goal and Joint Goal condition were (a) the task instruction participants received (as described above) and (b) the target color and shape. In the Individual Goal condition, the target always turned blue upon participants’ response (Fig. 3; left lower picture). In the Joint Goal condition, the target turned blue if participants responded to the opposite target as the co-actor and it turned green (with a star-shaped outline) if participants responded to the same target as the co-actor (Fig. 3; right lower picture).

As part of the general study instructions, participants were asked to turn off all distractions (e.g., music, phone, TV) and to perform the task diligently. At the end of the experiment, participants were asked whether they thought that the virtual partner was (a) a computer program, (b) another person, (c) another person’s pre-recorded performance, or (d) something else (see Table 1). Participants responded by pressing the corresponding letter key. Alternatively, participants could also press option (e) to indicate that they had not even thought about this issue.

**Table 1:** Questions participants received at the end of the experiment. The first and second question only appeared in Experiment 3; the last question was asked in all four experiments

Question	Response options
What was the joint goal in this game?	
	a) Moving to the OPPOSITE target as the yellow virtual partner.
	b) I don’t remember.
	*c) Moving to the SAME target as the yellow virtual partner.*
	d) I didn’t know there was a joint goal in this game.
The joint goal in this game was to move to the same target as the yellow virtual partner. Were you aware of this joint goal while performing the task?	
	a) Yes, I was aware of the joint goal. Whenever I moved to the same target as my partner, I knew that I was completing the joint goal.
	b) Yes, I was aware of the joint goal. However, I did not really consider it while performing the task.
	c) No, I was not aware of the joint goal. I read about it in the instruction text but then forgot it while performing the task.
	d) No, I was not aware of the joint goal. I didn’t know there was a joint goal in this game.
Finally, could you please tell us your thoughts about the yellow "virtual partner" in this game? Was it…	
	*a) a computer program?*
	b) another person?
	c) another person's pre-recorded performance
	d) other?
	e) Or have you not even thought about this?

Correct responses for the first and third question are marked by italics

#### Design

We used a 2 × 2 within-subjects design with the factors Target Location (Same, Different) – “Same” meant that participants responded to the same target as the co-actor and “Different” meant that they responded to the opposite target – and Condition (Individual Goal, Joint Goal).

The experiment was structured in four blocks with 120 trials each (480 trials total). Participants acted in half of the trials (and the co-actor in the other half); only the participants’ trials were analyzed (i.e., 240 trials). This resulted in 60 observations per cell of the 2 × 2 design. Each condition consisted of two consecutive blocks, with a short break in between. The order of conditions was counterbalanced across participants such that half started with the Individual Goal and the other half with the Joint Goal condition. After completing the first condition, participants were informed that there would be an important change in the second part of the experiment: they were told that from now on, they would be pursuing an individual/joint goal (depending on which condition they had started with). Before the start of the experiment, participants performed 32 practice trials to become familiarized with the task; they performed another 16 practice trials between conditions to become familiarized with the new goal.

The target location was randomized across trials, with the following constraints: (1) left and right locations were presented equally often as target within every set of eight trials; (2) the target appeared at the same location maximally six times in a row. Whether the participant’s cue appeared at the same or a different location as the co-actor’s previous target was randomized across trials. Same-/different-responses appeared equally often within every set of eight trials.

#### Data analysis

To test whether the presence of a joint goal shapes the social IOR, we conducted a 2 × 2 × 2 repeated-measures ANOVA with the within-subjects factors Target Location (Same, Different) and Condition (Individual Goal, Joint Goal), and the between-subjects factor Order (Joint Goal first, Joint Goal second), with response times (RTs) as dependent variable.^3^ RT was computed as the interval between the appearance of the cue and participants’ key press. The factor Order was included to check whether the temporal order in which participants encountered the two types of goals mattered, yet we had no a priori hypothesis about it.

To test whether any effect of joint goal on social IOR found in Experiment 1 would be similar in Experiments 2 and 3, we conducted between-Experiment comparisons. That is, after conducting the above-mentioned ANOVA that tests whether the social IOR is modulated by a joint goal, we tested to what extent the size of this modulation differs relative to Experiment 1 (joint goal *and* visual effect present) after either the joint goal (Experiment 2) or the visual effect (Experiment 3) has been removed. To capture the size of the modulation, we first computed the social IOR (as the difference in RT between same- and different-responses) per condition and then computed the difference in the magnitude of the social IOR between conditions. We then compared this difference value across experiments by means of an independent *t*-test.

In line with previous research (e.g., Welsh et al., 2005), the following trials were excluded from analysis: incorrect responses (wrong key pressed); responses that were unreasonably fast (< 100 ms; “anticipation errors”); responses that were unreasonably slow (> 1,000 ms; “inattention errors”). Also, whole data sets were excluded if participants evidently did not perform the task properly (< 90% accuracy rate) or if participants showed a large number of unreasonably slow or fast responses (> 10% of excluded trials).

For statistical inference, we used permutation-based ANOVAs. That is, the null distribution of the test statistics was estimated by repeatedly sampling permutations of the actual data under the assumption that there are no differences between the levels of our experimental factors (Kherad-Pajouh & Renaud, 2015). As effect size measures, we report generalized eta squared (η_G_^2^; Bakeman, 2005) and Cohen’s *d*.

### Results

In Experiment 1, 3.5% of all trials were excluded from analysis, in line with the data exclusion criteria reported above.

**Fig. 4:**
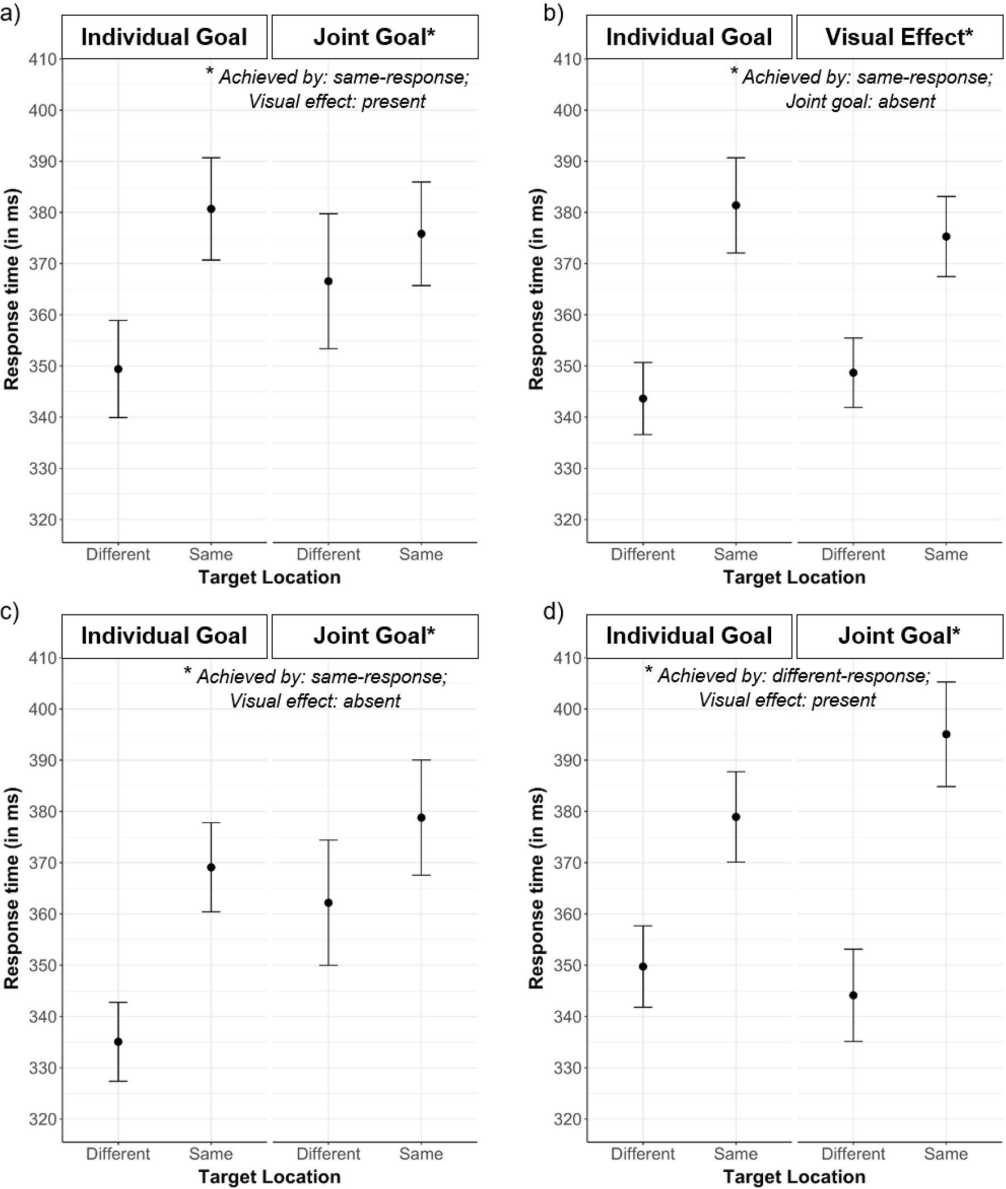
Response times for Experiments 1–4 (panels **a**–**d**, respectively), displayed as a function of Target Location (Different, Same) and Condition (Individual Goal, Joint Goal). Across experiments, same-responses were significantly slower than different-responses, reflecting the social inhibition of return (IOR). Importantly, the magnitude of the social IOR differed significantly between conditions. Error bars indicate standard errors

Figure 4a displays mean response times as a function of Target Location and Condition. Descriptively, participants responded more slowly to the target to which their co-actor had responded in the previous trial (henceforth “same-responses”) than to the opposite target (henceforth “different-responses”), displaying the expected social IOR response pattern. This was confirmed by a significant main effect of Target Location (*F*(1,32) = 20.98, *p* < .001, η_G_^2^ = .026). Importantly, there was also a significant interaction between Target Location and Condition (*F*(1,32) = 13.56, *p* = .001, η_G_^2^ = .008), indicating that the social IOR (reflected by the difference between same- and different-responses) was smaller in the Joint Goal (*M*_*diff*_ = 9 ms) compared to the Individual Goal condition (*M*_*diff*_ = 31 ms). This interaction can be ascribed mainly to slower different-responses in the Joint Goal (*M* = 367 ms) compared to the Individual Goal condition (*M* = 349 ms), see Fig. 4a. To quantify this difference in RT for different-responses, we report the effect size for the relevant pairwise comparison; Cohen’s *d* = 0.23. All other effects were not significant (all *p*s > .34).

In response to the last question about the identity of the co-actor, the majority of participants (26 out of 34, i.e., 76%) reported that they thought the co-actor was a computer program. Additionally, six participants (18%) believed that it was another person’s pre-recorded performance and two participants (6%) said that they had not even considered this issue.

### Discussion

The results of Experiment 1 showed that participants who took turns with a virtual co-actor in responding to targets on a computer screen were slower to respond to the target to which their co-actor had responded in the previous trial (same-response) than to the opposite target (different-response), as indicated by a significant main effect of Target Location. This difference between same-responses and different-responses reflects the well-established social IOR, i.e., the phenomenon of slowed return-responses to locations previously acted upon by another individual (Welsh et al., 2005). This result indicates – in line with previous research – that the social IOR occurs not only in live two-person settings where participants perform manual reaching responses, but also if participants interact with a co-actor who is not physically present (see Atkinson et al., 2018; Doneva & Cole, 2014; Nafcha et al., 2020b; but see also Skarratt et al., 2010) and if responses are keypresses (see Manzone et al., 2017; Nafcha et al., 2020b).

The crucial question was whether the magnitude of the social IOR would be affected by the goal participants pursued. Indeed, the significant interaction between Target Location and Condition showed that this was the case: The social IOR was smaller in the Joint Goal compared to the Individual Goal condition. While the achievement of the individual goal (coloring the target blue) depended only on participants’ own response, the achievement of the joint goal (coloring the target green) depended on the spatial relation between participants’ own and the co-actor’s previous response. Thus, the location of the co-actor’s previous response was goal-relevant. In line with earlier research showing that goal pursuit affects the allocation of spatial attention (Vogt et al., 2010), we predicted that, in the Joint Goal condition, participants should show delayed disengagement of attention from the goal-relevant (i.e., the co-actor’s previous) response location. The present results showed that the reduced social IOR in the Joint Goal condition was due to the fact that participants’ different-responses were distinctly slower in the Joint Goal compared to the Individual Goal condition (see Fig. 4a), suggesting that the prolonged attention on the goal-relevant location led to response inhibition for subsequent actions that were not directed towards the joint goal (compare Figs. 2a and 4a).

Taken together, the results of Experiment 1 provide initial support for the hypothesis that joint goals affect attentional orienting and hence the social IOR. However, in Experiment 1, the Joint Goal differed from the Individual Goal condition not only in terms of the goal participants pursued, but also in terms of the perceptual effect that was visible once that goal was achieved. If participants responded to the same location as the co-actor, the target turned blue in the Individual Goal condition and green in the Joint Goal condition. The green illumination was accompanied by a star-shaped green outline to increase its visual salience, in order to ensure that participants would not overlook the achievement of the joint goal. It is possible that this enhanced visual salience, rather than the joint goal itself, influenced participants’ attentional orienting.

## Experiment 2

Experiment 2 was conducted to test whether the modulation of the social IOR in Experiment 1 was due to the goal participants pursued or to the effect they observed. To disentangle conceptual goal from visual effect, we designed Experiment 2 such that it was perceptually identical to Experiment 1, yet differed conceptually in that there was no joint goal. This meant that in one of the two conditions, the target turned green whenever participants responded to the same location as the co-actor, but no conceptual framing was provided for this green effect.

If the modulation of the social IOR in Experiment 1 had been caused by the visually salient effect, then a similar pattern should be observed in Experiment 2. However, if the joint goal in Experiment 1 had caused the modulation, then no such modulation should occur in Experiment 2.

### Method

The methods in Experiment 2 were the same as in Experiment 1, with the following exceptions.

#### Participants

In Experiment 2, the analysis included 40 participants; no data sets were excluded (*M*_*age*_ = 23.65 years, *SD*_*age*_ = 4.91 years; 14 female, 24 male, one diverse, one unspecified). Twenty of these started with the Individual Goal condition and 20 with what was formerly known as the Joint Goal condition; the latter will henceforth be referred to as Visual Effect condition. Thirty-seven participants were right-handed, three were left-handed.

#### Stimuli and procedure

Instructions for the Visual Effect condition did *not* mention that participants shared a joint goal with the co-actor. Participants were merely informed that the target would turn green if they responded to the same location as the co-actor. Importantly, no meaning was attached to this effect.

#### Design

As in Experiment 1, one factor of the 2 × 2 within-subjects design was Target Location (Same, Different). The second factor was Condition, with the levels “Individual Goal” and “Visual Effect”.

### Results

In Experiment 2, 1.6% of all trials were excluded from analysis. As in Experiment 1, there was a significant main effect of Target Location (*F*(1,38) = 106.98, *p* < .001, η_G_^2^ = .111), demonstrating the social IOR response pattern (i.e., slower same- than different-responses); see Fig. 4b. This time, there was also a significant main effect of Order (*F*(1,38) = 5.73, *p* = .020, η_G_^2^ = .118), indicating that participants who started with the Visual Effect condition responded generally faster than those who started with the Individual Goal condition. There was also a significant interaction between Target Location and Order (*F*(1,38) = 5.63, *p* = .024, η_G_^2^ = .007), indicating that participants starting with the Individual Goal condition showed a larger social IOR overall. Importantly, as in Experiment 1, the interaction between Target Location and Condition was significant (*F*(1,38) = 10.72, *p* = .003, η_G_^2^ = .004), showing that the social IOR was smaller in the Visual Effect condition (*M*_*diff*_ = 27 ms) compared to the Individual Goal condition (*M*_*diff*_ = 38 ms). Again, this was due to slower different-responses in the Visual Effect condition (*M* = 349 ms) than in the Individual Goal condition (*M* = 344 ms); Cohen’s *d* = 0.12. All other effects were not significant (all *p*s > .24). When comparing the size of the social IOR modulation across Experiments 1 and 2 by means of an independent *t*-test, there was no significant difference (*t*(72) = 1.66, *p* = .101, Cohen’s *d* = 0.39), despite the descriptively larger modulation in Experiment 1.

As in Experiment 1, the majority of participants (28 out of 40, i.e., 70%) thought that the co-actor was a computer program. One participant (2.5%) thought it was another person and three (7.5%) that it was another person’s pre-recorded performance. Eight participants (20%) had not even considered this issue.

### Discussion

As expected, Experiment 2 replicated the social IOR observed in Experiment 1, as indexed by the significant main effect of Target Location. More importantly, the results of Experiment 2 helped to disentangle the influence of the goal participants pursued from the influence of the effect they observed. In particular, the results suggest that the presence of a visually salient effect is sufficient to influence participants’ attentional orienting, as indicated by the significant interaction between Target Location and Condition. That is, different-responses were slower in the Visual Effect condition compared to the Individual Goal condition, paralleling the findings from Experiment 1 where different-responses were slower in the Joint Goal compared to the Individual Goal condition (compare Fig. 4a and b).

The crucial difference between Experiments 1 and 2 was that participants in Experiment 2 were only aware that a certain effect (green illumination) would occur if they responded to the co-actor’s previous location, yet they received no instruction as to how to interpret this effect. Why did the green illumination by itself have an influence on participants’ attentional and response behavior? One possible explanation is that the green color served as a form of anticipated reward – firstly because of its arguably higher visual salience and secondly because it had a scarcity value since it appeared in only 12.5% of all experimental trials, in contrast to the co-actor’s yellow color (50%) and the participant’s individual blue color (37.5%). The prospect of earning a “green reward” if responding to the same location as the co-actor might have biased participants’ attention towards that location, resulting in comparatively slower responses to the opposite location. This would be in line with research showing that anticipated rewards guide selective attention such that relevant information is attended to optimize performance while the processing of less relevant information is suppressed (see reviews by Chelazzi et al., 2013, and Pessoa, 2015). Moreover, there is first evidence suggesting that rewards reduce the *non-social* IOR (Wang et al., 2022), which could be seen as support for the reward hypothesis advanced above.

The results of Experiment 2 showed that even if the visual effect is not explicitly framed in terms of a joint goal, its appearance is sufficient to cause a modulation of the social IOR, possibly because it serves a reward function. Alternatively, one can speculate that participants, even without instruction, might have interpreted the green color as an outcome of the complementary actions they performed with the co-actor (since it is obvious that yellow + blue = green). Thus, we cannot completely rule out that participants (implicitly or explicitly) perceived the Visual Effect condition as including a joint goal. Either way, an important open question that remained was whether and to what extent the concept of a joint goal by itself (i.e., without an accompanying visual effect) has an influence on the social IOR.

## Experiment 3

While in Experiment 1 participants were explicitly instructed to pursue a joint goal with the co-actor and they observed a visual effect upon achievement of that goal, participants in Experiment 2 only observed a visual effect yet there was no explicit mentioning of a joint goal. Consequently, Experiment 3 tested the influence of a joint goal while excluding any visual influence. Experiment 3 thus played a decisive role with regard to our main question of whether joint goals affect attentional orienting because, in contrast to Experiment 1, it focused on the joint goal as a purely internal state. This was done by comparing two conditions that were perceptually identical and only differed conceptually in terms of whether participants performed with a joint goal in mind or not.

If the modulation of the social IOR in Experiment 1 had been (partially) caused by the joint goal itself, then a similar pattern should be observed in Experiment 3. However, if the presence of a joint goal in Experiment 1 had no influence on the social IOR (but the modulation was caused solely by the visual effect), then no modulation should occur in Experiment 3.

### Method

The methods in Experiment 3 were the same as in Experiment 1, with the following exceptions.

#### Participants

In Experiment 3, 12 participants’ data sets (out of the total set of 40) were excluded from the analysis; five participants were excluded because they performed below 90% accuracy and seven participants because they gave an incorrect answer to the control question that verified whether they had paid attention to the task instructions (see below). Because the remaining sample of 28 participants did not guarantee sufficient power, we recruited 12 additional participants. Of these, again five were excluded (two because they performed below 90% accuracy and three because they failed the control question), resulting in a final sample of 35 participants (*M*_*age*_ = 26.03 years, *SD*_*age*_ = 5.86 years; 12 female, 22 male, one diverse). Nineteen of these started with the Individual Goal condition and 16 with the Joint Goal condition. Thirty-one participants were right-handed, three were left-handed, and one person did not specify handedness.

#### Stimuli and procedure

As in Experiment 1, participants were informed that the joint goal was to blend their own blue color with the co-actor’s yellow color by responding to the same target as the co-actor. In contrast to Experiment 1, the target did *not* illuminate in green when the joint goal was achieved; it simply turned blue as in the other trials.

Since in Experiment 3 there was no visual effect highlighting the achievement of the joint goal, it seemed necessary to ensure that participants did not simply forget about the joint goal while performing the task. To this end, a short reminder of the task instruction was displayed two times throughout each block in the Joint Goal condition (i.e., once every 40 trials). Moreover, at the end of the experiment, participants were asked to respond to two additional control questions. The first question was simply “What was the joint goal?” (see Table 1 for response options). This question served as a test of whether participants had actually paid attention to the task instructions. After responding, participants were informed about the correct answer. They were then asked whether they had been aware of the joint goal while performing the task (see Table 1).

#### Data analysis

Participants who did not know what the joint goal was and thus answered the corresponding control question (see above) incorrectly were excluded from the analysis because they had evidently not paid attention to the task instructions.

### Results

In Experiment 3, 4.5% of all trials were excluded from analysis. As in Experiments 1 and 2, there was a significant main effect of Target Location (*F*(1,33) = 53.25, *p* < .001, η_G_^2^ = .046), showing the social IOR (see Fig. 4c). This time, there was also a significant main effect of Condition (*F*(1,33) = 6.62, *p* = .014, η_G_^2^ = .023), indicating that participants responded generally faster in the Individual Goal (*M* = 352 ms) than in the Joint Goal condition (*M* = 371 ms), and a significant interaction between Target Location and Order (*F*(1,33) = 4.31, *p* = .046, η_G_^2^ = .004), indicating that participants starting with the Individual Goal condition showed a smaller social IOR overall. Importantly, as in Experiments 1 and 2, the interaction between Target Location and Condition was also significant (*F*(1,33) = 6.89, *p* = .012, η_G_^2^ = .005), showing that the social IOR was smaller in the Joint Goal (*M*_*diff*_ = 17 ms) compared to the Individual Goal condition (*M*_*diff*_ = 34 ms). Again, this was due to slower different-responses in the Joint Goal (*M* = 362 ms) than in the Individual Goal condition (*M* = 335 ms); Cohen’s *d* = 0.41. When comparing the size of the social IOR modulation across Experiments 1 and 3 by means of an independent *t*-test, there was no significant difference (*t*(67) = 0.52, *p* = .604, Cohen’s *d* = 0.13).

Finally, there was also a significant three-way interaction between Target Location, Condition and Order (*F*(1,33) = 6.65, *p* = .014, η_G_^2^ = .004). All other effects were not significant (all *p*s > .44). To follow up this three-way interaction, we performed two 2 × 2 ANOVAs with the factors Target Location and Condition, separately for each Order; see Fig. 1 in the Online Supplementary Material. When participants started with the Individual Goal condition, results paralleled our main analysis: There was a significant main effect of Target Location (*F*(1,18) = 12.62, *p* = .003, η_G_^2^ = .021), a significant main effect of Condition (*F*(1,18) = 6.66, *p* = .020, η_G_^2^ = .032), and, most importantly, a significant interaction between these two factors (*F*(1,18) = 9.58, *p* = .005, η_G_^2^ = .015). However, when participants started with the Joint Goal condition, results differed in that there was only a significant main effect of Target Location (*F*(1,15) = 51.74, *p* < .001, η_G_^2^ = .092), yet no other significant effects (all *p*s > .26). Thus, the social IOR was present regardless of the order of conditions, yet it was only affected by the joint goal if participants had encountered the individual goal first.

As in Experiments 1 and 2, the majority of participants (27 out of 35, i.e., 77%) thought that the co-actor was a computer program. One participant (3%) thought it was another person and three (9%) that it was another person’s pre-recorded performance. Four participants (11%) had not even considered this issue. As confirmed by the first control question, all participants knew that the joint goal was to move to the same location as the co-actor.^4^ In response to the second control question, the majority (23 out of 35, i.e., 66%) said that they were aware of the joint goal and actively considered it while performing the task, while another 11 participants (31%) said that they were aware of the joint goal but did not actively consider it. Only one participant reported not being aware of the joint goal while performing the task.

### Discussion

Besides replicating the social IOR, Experiment 3 demonstrated that the joint goal by itself (i.e., without an accompanying visual effect) influenced participants’ attentional orienting and hence modulated the social IOR, as indicated by the significant interaction between Target Location and Condition. As in Experiments 1 and 2, the reduced social IOR was mainly due to participants’ slower different-responses in the Joint Goal compared to the Individual Goal condition (see Fig. 4c). Importantly, the reduction of the social IOR in the Joint Goal condition cannot be attributed to any perceptual differences because the physical and temporal characteristics of the stimuli were identical across conditions. The two conditions differed only in terms of whether participants performed with a joint goal in mind or not.

In contrast to Experiment 1, the joint goal in Experiment 3 only affected participants’ behavior if they had encountered the individual goal first. The likely reason for this order effect is that participants in Experiment 3 first needed to experience the individual goal before they could fully make sense of the joint goal instruction. This is because the instruction was more difficult to grasp because the Joint Goal condition did not differ perceptually from the Individual Goal condition – and therefore might have only made sense if it could be seen against the background of the Individual Goal condition. Participants’ general difficulty to conceive of a joint goal in the absence of any external feedback about the achievement of that goal would also explain the relatively high number of excluded data sets, with more exclusions for participants starting with the Joint Goal compared to the Individual Goal condition.

Importantly, on average, participants in Experiment 3 showed a similar response behavior in the Joint Goal condition as those in Experiment 1, as indicated by the comparable magnitude of the social IOR modulation in Experiments 1 and 3 (22 ms vs. 17 ms).

## Experiment 4

When participants pursued a joint goal in Experiments 1 and 3, the social IOR was reduced compared to when they pursued an individual goal, suggesting that joint goals modulate the social IOR. However, it is not clear whether this modulating effect is specific for complementary actions directed to the same location (as in Experiments 1 and 3) or whether it applies to complementary actions more generally. What about interactions that require individuals to act upon *different* locations? Consider, again, two friends painting a landscape together. One of them applies blue paint to the bottom of the canvas (depicting the sea), before the other applies yellow to the top (depicting the sun light). They achieve their joint goal by acting in complementary ways at different locations.

To address the above question, we modified the joint goal in Experiment 4 such that it was achieved if participants responded precisely *not* to the co-actor’s but to the opposite location. If, as suggested by the results of Experiments 1 and 3, a goal-relevant location leads to delayed disengagement of attention from that location and response inhibition to other, non-goal locations, then the same attention modulation should occur in Experiment 4. Specifically, this means that participants in Experiment 4 should show delayed disengagement of attention from the location opposite to the co-actor’s response and hence slower responses to the same location. If, however, the modulation of the social IOR observed in Experiments 1 and 3 was specific to complementary actions directed to the *same* location, then no such modulation should occur in Experiment 4.

### Method

The methods in Experiment 4 were the same as in Experiment 1, with the following exceptions.

#### Participants

In Experiment 4, six participants’ data sets were excluded from the analysis because performance was below 90% accuracy, resulting in a final sample of 34 participants (*M*_*age*_ = 24.29 years, *SD*_*age*_ = 5.21 years; nine female, 24 male, one diverse). Seventeen of these started with the Individual Goal condition and 17 with the Joint Goal condition. Twenty-eight participants were right-handed, six were left-handed.

#### Stimuli and procedure

Participants were informed that the joint goal was to color the two targets *one after the other*. That is, in order to reach the joint goal, participants needed to respond not to the location to which the co-actor had responded in the previous trial but to the opposite one. Whenever participants responded to the opposite target, that target turned green.

### Results

In Experiment 4, 3.3% of all trials were excluded from analysis. As in all previous experiments, there was a significant main effect of Target Location (*F*(1,32) = 172.80, *p* < .001, η_G_^2^ = .139), again replicating the social IOR (see Fig. 4d). There was also a significant interaction effect between Target Location and Condition (*F*(1,32) = 16.65, *p* < .001, η_G_^2^ = .012). This time, however, the social IOR was larger in the Joint Goal (*M*_*diff*_ = 51 ms) compared to the Individual Goal condition (*M*_*diff*_ = 29 ms). This was due to slower same-responses in the Joint Goal (*M* = 395 ms) than in the Individual Goal condition (*M* = 379 ms); Cohen’s *d* = 0.28. This pattern was mirror-inverted compared to Experiments 1–3. All other effects were not significant (all *p*s > .10).

As in all previous experiments, the majority of participants (24 out of 34, i.e., 70.5%) thought that the co-actor was a computer program. One participant (3%) thought it was another person and seven (20.5%) that it was another person’s pre-recorded performance. Two participants (6%) had not even considered this issue.

### Discussion

The results of Experiment 4 showed that the pattern observed in Experiments 1 and 3 was not specific to complementary actions directed to the same location but also applies to complementary actions directed to different locations. In particular, while participants in Experiments 1 and 3 reached the joint goal by responding to the same location as the co-actor (same-response), participants in Experiment 4 did so by responding to the opposite location (different-response). Consequently, while comparatively slower *different*-responses were observed in the Joint Goal condition of Experiments 1 and 3, slower *same*-responses were observed in Experiment 4. The latter pattern suggests that when participants in Experiment 4 observed the co-actor’s response to location X, their attention initially shifted reflexively to location X but then disengaged and oriented towards the opposite location Y, in line with the typical attentional orienting pattern (e.g., Klein, 2000). Importantly, if participants were then cued to respond to location X, they were presumably slower to disengage their attention from Y because Y was goal-relevant (compare Vogt et al., 2010). The slower return-responses that characterize the social IOR were thus reinforced in this case, resulting in an even larger difference in response times between same- and different-responses (see Fig. 4d).

Supporting the results of Experiments 1 and 3, Experiment 4 provided further evidence for our original hypothesis that joint goals affect attentional orienting and hence the social IOR.

## General discussion

The present study addressed the question of whether pursuing a joint goal with another agent shapes the way in which that agent’s action shapes our own. While previous research on the same topic focused on cases where two individuals perform complementary actions with different kinematic parameters (e.g., Clarke et al., 2019; Della Gatta et al., 2017; Sacheli et al., 2012, 2013, 2018), the present study focused on the spatial location these actions are directed at. In particular, we examined how the spatial relation between two individuals’ actions influences their attentional and response behavior – and how this influence is modulated by the goal individuals pursue. Specifically, we tested whether the magnitude of the social inhibition of return (social IOR) – a phenomenon characterized by slower return responses to locations previously acted upon by another agent – is reduced if two agents pursue a joint goal.

To this end, we designed a computerized social IOR task where participants responded to one of two target locations on a computer screen, taking turns with a virtual co-actor. Participants either pursued an individual goal or they performed complementary actions with the co-actor, in pursuit of a joint goal. Specifically, participants either aimed to color the target with their own blue color (Individual Goal condition) or to blend their own blue with the co-actor’s yellow color, thereby turning the target green (Joint Goal condition). Participants achieved the joint goal (in Exp. 1 and 3) if they responded to the same target location as the co-actor in the previous trial. In line with research on top-down attention (Vogt et al., 2010), we predicted that the presence of a joint goal would affect participants’ attentional orienting and subsequent response behavior, and hence the social IOR. In particular, participants should show delayed disengagement of attention from the goal-relevant (i.e., the co-actor’s previous) location, which in turn would likely lead to a response *inhibition* for the opposite, non-goal location (different-responses), or, potentially, to a *facilitation* for the goal-relevant location (same-responses). Both would effectively result in a reduced social IOR (see Fig. 2). We conducted a series of four experiments to test whether joint goals affect attentional orienting and if so, whether this leads to response inhibition or facilitation.

Experiment 1 showed that the social IOR was significantly modulated when participants shared a joint goal with the co-actor. This modulation was mainly due to slower different-responses in the Joint Goal compared to the Individual Goal condition, indicating response *inhibition* for actions that were not goal-relevant. Experiments 2 and 3 tested whether the reduction of the social IOR observed in Experiment 1 was actually due to the goal participants pursued or to the visual effect they observed upon goal achievement (i.e., a green target illumination). Results showed that an (observable) visual effect by itself is sufficient to influence the social IOR, possibly because it is perceived as a reward or because it is automatically construed as a joint outcome (Exp. 2). Yet an (unobservable) joint goal by itself is similarly influential (Exp. 3). Experiment 4 demonstrated that the observed modulation occurs not only for complementary actions directed to the same location (Exps. 1–3), but also for complementary actions directed to different locations. Together, the results of Experiments 1–4 show that attentional orienting, and hence the social IOR, is affected by joint goals. Our study thus supports and extends previous research on motor interference effects which has shown that the extent to which a co-actor’s observed action interferes with one’s own executed action is modulated by whether these actions are directed towards an individual or a joint goal (Clarke et al., 2019; Della Gatta et al., 2017; Sacheli et al., 2012, 2013, 2018).

One aspect that deserves further attention is the direction of the modulation observed in the present study. Specifically, we observed that the presence of a joint goal primarily had an inhibitory effect on responses that were not goal-relevant – rather than a facilitatory effect on goal-relevant responses. Considering real-world scenarios where one individual aims to achieve a joint goal by returning to the same spatial location another individual just acted upon (e.g., one individual pours milk into a cup after another poured in the tea), one might think that slowed returns are not beneficial for such complementary joint actions – and thus, one might have expected that the pursuit of joint goals leads to a speed-up of these returns. However, the present results show that return-responses are only minimally (or not at all) faster in the presence of a joint goal. This suggests that the inhibitory process at the core of the social IOR is very persistent and not easily affected. Of course, speeding an action up is usually more difficult than slowing it down – so the present result pattern might also reflect a ceiling effect in the sense that return-responses were already as fast as they could be. Even though we did not observe faster return-responses to the goal-relevant location, we still saw a clear modulating effect of the joint goal, indicated by the slower responses to the non-goal location. This suggests that the possibility of achieving a joint goal was prioritized over the achievement of an individual goal, which in turn influenced attention allocation and subsequent response behavior. This result is consistent with previous research showing that the prioritizing of goals affects spatial attention such that, when multiple goals exist, attention is oriented to events relevant to the priority goal (Vogt et al., 2011).

### The sociality of the social IOR

Even though the present study did not primarily aim at contributing to the debate on how social the social IOR actually is (see, e.g., Atkinson et al., 2018; Doneva & Cole, 2014; Doneva et al., 2017; Nafcha et al., 2020a, b; Skarratt et al., 2010), it still makes a relevant contribution in the following respects. First, it reaffirmed previous findings showing that the social IOR occurs not only in live two-person settings but also if participants interact with a co-actor who is not physically present (compare Atkinson et al., 2018; Doneva & Cole, 2014; Nafcha et al., 2020b). In previous studies in which the co-actor was absent, participants typically received at least some information about whom they were (supposedly) interacting with, for example, they were able to observe a video feed or animated image sequence of the co-actor (Atkinson et al., 2018; Skarratt et al., 2010) or they were first introduced to a co-actor who was then seated in another room (Nafcha et al., 2020b). In the present study, however, participants were only informed that they were performing the task together with a “virtual partner” yet they did not receive any further information about the co-actor’s identity. Moreover, in contrast to previous studies, the present study was not conducted in a laboratory setting but online. Arguably, the remote online setting might have created a more abstract and less social atmosphere compared to a lab setting. Indeed, the majority (~ 73%) of all participants in the present study believed that the co-actor was a computer program (which was the truth); thus, they were aware that this was not a live interaction with another human being. The fact that the social IOR occurs even in remote human-computer interactions could be seen as support of previous findings which suggest that the “social” IOR only meets a minimal threshold to be considered a social phenomenon (compare Atkinson et al., 2018). However, one could just as well argue that in today’s world, where virtual interactions have become increasingly more common and where artificial intelligence is quickly taking root in everyday life, it seems difficult to classify a phenomenon as intrinsically “not social” solely because it extends to human-AI interactions. After all, mechanisms of social cognition (e.g., mental state attribution) may be evoked not only in interactions with natural human agents but also in those with artificial agents (e.g., Hortensius & Cross, 2018; Hortensius et al., 2018; Powell & Michael, 2019; Wahn & Kingstone, 2021; Wykowska et al., 2016).

Moreover, the present findings suggest that participants perceived the interaction as social to the extent that the idea of pursuing a joint goal together with the co-actor (a) seemed feasible and (b) differed from pursuing an individual goal. The fact that participants’ attentional orienting depended on the pursued goal clearly indicates that having a (social) joint goal in mind made a difference – a difference that cannot be attributed to any external factors such as stimulus attributes because those did not differ (see Experiment 3). Converging evidence comes from a study by Gobel et al. (2018) who demonstrated, also using a social IOR paradigm, that attentional orienting is tuned to the social relevance of a cue. In particular, Gobel et al. showed that the magnitude of the social IOR was larger when participants believed that the cue they observed was generated by a human instead of a computer. In that study, just as in the present one, differences in participants’ attention cannot be attributed to the physical environment but to the way in which participants mentally represented that environment (Gobel et al., 2018; also see Richardson & Gobel, 2015). Further recent studies by the same authors (Gobel & Giesbrecht, 2020; Tufft & Gobel, 2022) also indicated that social top-down information can affect low-level spatial orienting.

Finally, it is important to clarify the claims that can be made based on the present findings, and, even more importantly, the claims that cannot be made. As already mentioned at the outset, the goal of the present study was to address the question of whether pursuing a joint goal with another agent shapes the way in which that agent’s action shapes our own. The question was not whether the social nature of an interaction, more generally, shapes our own actions. To put the focus on the goal pursuit, we kept the nature of the interaction constant while varying only participants’ goal. Thus, in both the Individual Goal and the Joint Goal condition, participants took turns with a so-called “virtual partner”. What varied was whether, within this interaction, participants pursued an individual or a joint goal. Thus, regardless of whether the interaction was perceived as more or less social – i.e., whether participants believed to be interacting with a computer or a human – the perceived nature of the interaction stayed constant across conditions. Only the type of goal varied. Hence, there are no claims to be made about the effect of the sociality of the interaction as such. In fact, we intentionally decided not to deceive participants (e.g., by making them believe they were interacting with another human) but to keep the description of the interaction partner as neutral as possible by calling it a “virtual partner”. We acknowledge explicitly (see above) that most participants believed to be interacting with a computer and thus were aware that this was not a human-human social interaction. Moreover, based on the results from Experiment 2, we also acknowledge that our main finding (a modulation of the social IOR) also occurs if the task is not explicitly framed in terms of a joint goal but participants merely observe a salient visual effect as a result of their complementary action. This might be because the visual effect is perceived as a (non-social) reward. However, we also find (in Experiment 3) that the modulation of the social IOR occurs if the two conditions are perceptually identical and the only difference is whether the task is framed in terms of a joint goal or not. This shows that the framing in terms of a joint goal does have an influence. Together, our experiments suggest that the goal-relevance of another agent’s action – whether this goal is perceived as intrinsically social or not – shapes our own actions. What made another’s action goal-relevant, in our study, was the fact that participants could add their own action in a complementary way, which resulted in a certain visual effect (or was merely construed as resulting in the achievement of a joint goal, see Exp. 3).

### Goals and the social IOR

While the present study focused on the impact of *joint* goals on the social IOR, a considerable amount of previous studies investigated the impact of action goals more generally. In particular, 14 experiments published in five different articles (Cole et al., 2012, 2018; Janczyk et al., 2016; Ondobaka et al., 2012, 2013) have addressed the question of whether the social IOR is sensitive to goals (see Cole et al., 2019). Whereas in the standard social IOR procedure, participants’ only goal is to reach towards and touch the target, participants in the above-mentioned experiments were given an additional task to be performed *at* the target. Accordingly, the authors defined a goal as the “terminal component of a reaching action” (Cole et al., 2012), i.e., the task to be completed *after* the target has been reached. Goal compatibility was manipulated such that a participant either performed the same or a different task as the co-actor in the previous trial. For example, participants would be required to reach towards the target and pick up a pen to either write with it or to use its opposite end as an eraser (Cole et al., 2012). Using this and similar manipulations of the ‘endpoint goal’, it was tested whether the compatibility of goals mediates the social IOR. The results of all but one experiment (see Ondobaka et al., 2012) showed that this was not the case, suggesting that one’s own action is shaped by the location yet not by the goal another agent’s observed action is directed at. While this finding is certainly informative with regard to the nature of the social IOR and its underlying mechanisms (see Cole et al., 2018, 2019), it does not lead to direct conclusions regarding *joint* goals.

One should note, though, that the study by Cole et al. (2018) differs from the above studies in that it actually included one experiment with a joint goal condition. However, the conception of the joint goal differed crucially from the one in the present study. Specifically, in Experiment 5 of Cole et al.’s study, the goal was manipulated such that participants were asked to collaborate with a confederate on a task in one block of trials and to perform the same task alone in another block. The task was to complete a dot-to-dot drawing. In each trial, participant (and confederate) would reach to the target location to pick up a pen and connect two dots such that at the end of the block, the drawing would be completed and the joint goal achieved. However, the joint goal could only be achieved in the collaborative block where the confederate contributed to the drawing. Results showed that this type of joint goal manipulation did not affect the social IOR. There are several differences between that study and the present one. First, in Cole et al., the joint goal was reflected in a collaborative task that was completed throughout the entire block of trials whereas in the present study, the joint goal was contingent on the two agents’ trial-to-trial contributions. Furthermore, in Cole et al., it did not matter to which target location participant and co-actor reached, as long as they connected two dots at the respective location. Thus, the spatial relation between agents’ actions (i.e., whether they subsequently reached to the same target or not) did not play a role. In the present study, the spatial relation was decisive, as it determined whether the joint goal was achieved or not. Thus, the differing results of the two studies can be attributed to the different ways of operationalizing a joint goal and hence they do not actually conflict.

We agree with Cole and colleagues that it remains a big challenge to define a joint goal (or a joint action, for that matter; see Paternotte, 2014) in the laboratory because goals can be defined at various levels of specificity. It is true that in the broadest sense, participants in any joint action experiment always share the goal of participating in a particular joint action task (Cole et al., 2018). This is the level at which goals were manipulated in the study by Cole et al. (2018) where participants performed a joint action (complete the drawing together) in one condition and an individual action (complete the drawing alone) in the other condition. In the present study, goals were manipulated at a more specific level, namely at the level of action intentions. It would be worthwhile to further investigate how a joint goal must be conceptualized for it to affect the interplay between observed and executed actions in this specific and in other joint action scenarios.

### Open questions and future directions

The present findings raise several questions for future research to address. One question is whether the influence of joint goal pursuit on the social IOR that we observed in a human-computer interaction might be even stronger in a real interpersonal interaction. The influence might be stronger simply because interactive engagement, interest and commitment should be higher when interacting with another human than with a virtual agent (e.g., Powell & Michael, 2019; Raij et al., 2007). This prediction could be tested by replicating the present study in a typical lab setting with two participants performing manual reaching actions (e.g., Welsh et al., 2005; see Fig. 1a).

Another open question is whether the influence of a joint goal depends on the outcome associated with achieving that goal. In particular, one could compare the simple visual effect used in the present study with joint goals that are associated with scored points or even monetary reward. In addition, one could introduce a condition without a joint goal but with monetary reward only. This way, one could test whether joint goals and rewards have similar effects on attentional orienting (as hypothesized earlier) and whether these effects might be additive.

A further interesting question concerns the difference between dictated versus free choice responses. Specifically, one could contrast the present study, where participants’ responses were dictated by a cue, with a situation where participants can freely choose which location to respond to (and thus whether to achieve the joint goal or not). In fact, earlier research showed that participants, if given the choice, are less likely to respond to their co-actor’s previous location than to the opposite location (Cole et al., 2015). Testing whether the presence of a joint goal alters this tendency would show if joint goal pursuit modulates solely people’s attentional preferences or if it also modulates their choice preferences.

Finally, one could translate the present study into a classical cueing paradigm where responses to previously attended locations are facilitated rather than inhibited (Posner & Cohen, 1984). As in the present study, the joint goal would be achieved by responding to the same (i.e., cued) location as the co-actor. The question is whether the prospect of achieving a joint goal would result in increased facilitation for the cued location and/or increased inhibition for the uncued location, i.e., whether one would see a similar, yet inverse response pattern as in the present study.

## Conclusion

When pursuing a joint goal, people often attend and respond to the same object or spatial location in complementary ways. In the present work, we examined whether joint goal pursuit modulates the extent to which another agent’s perceived action shapes our own action, specifically when both actions are directed to the same location. While previous research has shown that the *spatial* relation between agents’ actions (i.e., whether they are directed towards the same or different locations) influences attentional and response behavior, we have shown here that the *social* relation between agents’ actions (i.e., whether they are directed towards the same or different goals) has a modulating effect on that influence. In particular, we demonstrate that introducing a joint goal – even in an artificial social context without a real human co-actor – guides individuals’ attentional orienting. Taken together, our findings extend previous research on interpersonal perception-action effects, showing that the way another agent’s perceived action shapes our own depends on whether we share a joint goal with that agent.

## Supplementary Information

Below is the link to the electronic supplementary material.

Supplementary file1 (DOCX 114 KB)

## Data Availability

All data and stimuli are publicly available via the Open Science Framework: https://osf.io/4yfd2/?view_only=91af170d9953437bb9718a62ed5898af.

## Footnotes

1 It is worth noting that work by Cole et al. (2018) actually included a joint goal manipulation. However, their conception of a joint goal differed crucially from that used in the present study. We return to this point in the *General discussion*.

2 The choice for a computerized task was not theoretically motivated but mainly a pragmatic decision due to closed laboratories during the COVID-19 pandemic.

3 One should of course consider that temporal precision for response times is not as good in *online* studies as in *lab-based* studies. However, recent research (Anwyl-Irvine et al., 2021; Bridges et al. 2020) tested different popular packages and platforms for online studies (incl. PsychoPy, which was used for the present study) and found that modern web platforms provide reasonable precision for manual response time.

4 Participants who answered this question incorrectly were not included in the analysis.

## Code availability

The code will be readily provided upon request.
