# Supplementary material for: Attention allocation in complementary joint action: How joint goals affect spatial orienting
Source: Atten Percept Psychophys. 2023 Sep 8;86(5):1574–93. doi: 10.3758/s13414-023-02779-1 (PMC11557662; doi:10.3758/s13414-023-02779-1)
Supplement: Supplementary file 1 — Supplementary file1 (DOCX 114 KB) [file 13414_2023_2779_MOESM1_ESM.docx]

# Supplementary material

**Results Exp. 3:** Three-way interaction between Target Location, Condition and Order


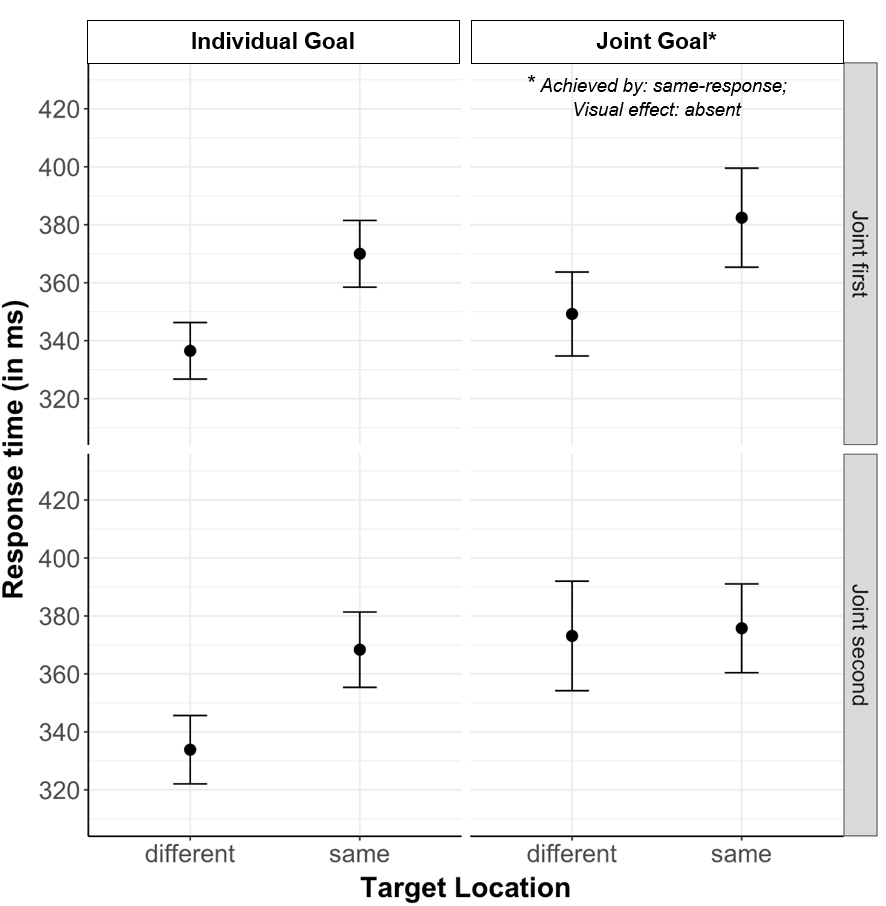


**Figure 1.** The plot illustrates the results of the 2 × 2 × 2 repeated measures ANOVA with the within-subjects factors Target Location (Different, Same) and Condition (Individual Goal, Joint Goal), and the between-subjects factor Order (Joint Goal first, Joint Goal second). Response times are displayed as a function of Target Location and Condition; the upper panels show the data of those participants who started with the Joint Goal condition and the lower panels show the data of those who started with the Individual Goal condition. The magnitude of the social IOR differed significantly as a function of Condition, yet only for participants who encountered the individual goal first (lower panels). Error bars indicate Standard Errors.
